# Construction of an miRNA-Regulated Pathway Network Reveals Candidate Biomarkers for Postmenopausal Osteoporosis

**DOI:** 10.1155/2017/9426280

**Published:** 2017-10-11

**Authors:** Min Shao

**Affiliations:** Department of Orthopedics, Third Affiliated Hospital of Guangzhou University of Chinese Medicine, Guangzhou, Guangdong Province 510240, China

## Abstract

We aimed to identify risk pathways for postmenopausal osteoporosis (PMOP) via establishing an microRNAs- (miRNA-) regulated pathway network (MRPN). Firstly, we identified differential pathways through calculating gene- and pathway-level statistics based on the accumulated normal samples using the individual pathway aberrance score (iPAS). Significant pathways based on differentially expressed genes (DEGs) using DAVID were extracted, followed by identifying the common pathways between iPAS and DAVID methods. Next, miRNAs prediction was implemented via calculating TargetScore values with precomputed input (log fold change (FC), TargetScan context score (TSCS), and probabilities of conserved targeting (PCT)). An MRPN construction was constructed using the common genes in the common pathways and the predicted miRNAs. Using false discovery rate (FDR) < 0.05, 279 differential pathways were identified. Using the criteria of FDR < 0.05 and |log⁡FC| ≥ 2, 39 DEGs were retrieved, and these DEGs were enriched in 64 significant pathways identified by DAVID. Overall, 27 pathways were the common ones between two methods. Importantly, MAPK signaling pathway and PI3K-Akt signaling pathway were the first and second significantly enriched ones, respectively. These 27 common pathways separated PMOP from controls with the accuracy of 0.912. MAPK signaling pathway and PI3K/Akt signaling pathway might play crucial roles in PMOP.

## 1. Introduction

Estrogen plays a crucial role in keeping healthy bones. Estrogen deficiency has significantly negative effect on bone cell functions [1]. Postmenopausal osteoporosis (PMOP) is believed to result directly from the decreased endogenous estrogen in menopausal women [2, 3]. PMOP is characterized by the severe loss of bone mass in the vertebrae and long bones. Globally, thousands of women over 50 years of age are influenced by PMOP which causes an increase of economic and societal burden recently. Therefore, studying PMOP-related molecular mechanisms is urgently needed.

In recent years, previous studies have demonstrated that there are powerful genetic effects on the etiology of osteoporosis (OP) in postmenopausal women [4–6]. For example, polymorphisms in a set of genes have been found to be related to PMOP women in China, including* ESR2* [7],* HLA-B *[8], and* OPG* [9]. Nevertheless, the etiology of PMOP still remains limited. MicroRNAs (miRNAs), as a kind of small noncoding RNAs of 22–25 nucleotides, have been implicated to exert indispensable regulatory functions in gene expression in animal kingdoms [10]. miRNAs have also been reported to play important roles in the regulation of biological processes, such as cell apoptosis, proliferation, and differentiation [11, 12]. Growing evidence has demonstrated that miRNAs have an important role in regulating bone mass. In recent years, the role of miRNAs in the progress of OP has gained interest. Numerous studies have demonstrated that the dysregulation of miRNAs is associated with OP. miRNA-2861 has been found to affect osteoblast differentiation, contributing to OP in the femur via its effects on osteoblasts [13]. Moreover, miRNA-34c has been implicated to enhance the osteoclastogenesis through regulating the genes involved in the Notch signaling pathway [14]. Moreover, Chen et al. [15] have demonstrated that miRNA-125b is significantly overexpressed in PMOP, and miRNA-125b may be a potentially noninvasive biosignature for PMOP. Similarly, Xiao et al. [16] have suggested the role of miRNA-129-5p in osteoblast differentiation and bone homeostasis. These findings suggest that miRNAs have potential effects on the pathways in the progression of OP and can thereby affect the treatment of OP. However, most of the studies have focused on only one or a few miRNAs/pathways using limited samples. Of note, little is known about specific pathways regulated by miRNAs during the development of PMOP.

Hence, we aimed to detect the risk pathways regulated by PMOP-related miRNAs through establishing an miRNA-regulated pathway network (MRPN). Then, we analyzed the topological characteristics of the MRPN, and we also determined how the miRNAs regulated PMOP risk pathways. The results of our study may provide a novel viewpoint with respect to the mechanism and treatment of PMOP.

## 2. Methods

**Figure 1:**
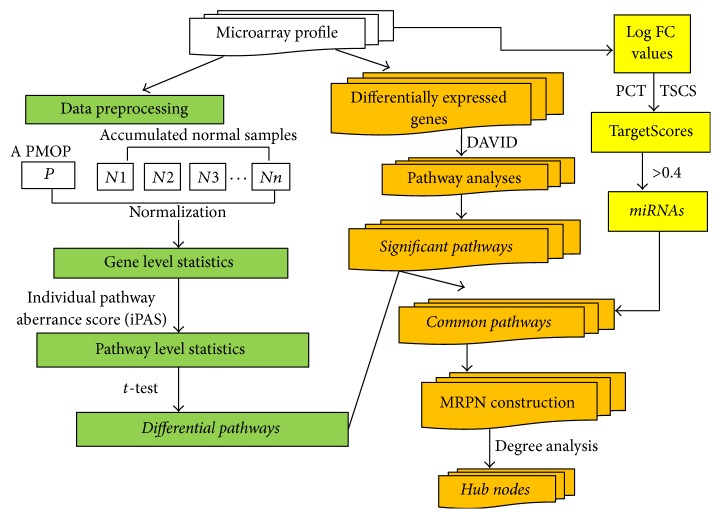
The flowchart showing the workflow step-by-step.

The current analysis was comprised of the following steps-data selection (microarray profile, pathway data): pathway identification through calculating gene-level statistics and pathway-level statistics based on the accumulated normal samples using individual pathway aberrance score (iPAS), finding differentially expressed genes (DEGs) and significant pathways based on DEGs using DAVID software, identification of the common pathways of these two methods, miRNAs prediction based on the common genes via calculating TargetScores, MRPN construction, and the topological properties for MRPN. The detailed information of each step was described in Figure 1.

### 2.1. Data Availability

To reveal the molecular mechanisms of PMOP, microarray analyses of monocytes were performed using Affymetrix HG-133A arrays in 80 females, including 40 pre- and 40 postmenopausal subjects. These data were deposited in the E-GEOD-56815 of the ArrayExpress database [14]. Probe IDs owning concentrated expression level were transformed into human gene symbols. Duplicated genes in matrix were eliminated. Overall, we obtained 8450 genes.

### 2.2. Collection of Pathway Data

All biological pathways of humans were retrieved from REACTOME database (http://www.reactome.org/) [17]. Then, several pathways were filtered out when the gene set size of these pathways was larger than 100. In addition, the pathways with the intersection of 0 between genes enriched in the pathways and 8450 genes identified in the present analysis were also discarded. Ultimately, 1078 pathways were reserved for further analysis.

### 2.3. Gene-Level Statistics

Herein, accumulated premenopausal samples were used as a reference and the expression level of genes was computed by comparing one postmenopause sample with many accumulated premenopause samples according to robust multichip average [18]. In detail, we firstly normalized the genes in all the premenopause subjects for the reference one by one to the reference, and then we calculated the mean value as well as standard deviation (SD) of the expression level. For a single postmenopause sample, quantile normalization was conducted after integrating the individual postmenopause sample with all reference samples. Assuming the genes having several probes, gene expression value was computed via averaging probe expression value. Gene-level statistics of each gene in an individual postmenopause subject was standardized according to the mean value and SD of the reference genes.

### 2.4. Pathway-Level Statistics

We used the average *Z* method to calculate the pathway activity. Specifically, the gene-level statistics of all genes in each pathway were measured and summarized, following by transforming the mean value of gene-level into the pathway statistics of this pathway. The pathway statistics was calculated using the following formula:

(1) $$\begin{array}{c} iPAS=\frac{\sum_{i}^{m}Z_{i}}{m}. \end{array}$$

In this formula, iPAS is the expression status of a pathway, *Z*_*i*_ represents the gene-level statistics of *i*th gene, and *m* denotes the count of genes enriched in this given pathway.

### 2.5. Identification of Differential Pathways

With the goal of analyzing the changes of pathways in PMOP and control samples, Gitools [19] was utilized to construct the cluster heatmap of pathways. *t*-test was used to measure the pathway statistics of each pathway in all samples of the two groups. All the premenopause samples for the reference were compared one by one with reference to output the null distribution of pathway statistics. On the basis of the comparison between an individual postmenopause sample and the null distribution, we obtained the raw *P* value. Subsequently, the *P* values were corrected for multiple hypothesis testing based on false discovery rate (FDR) control using Benjamini and Hochberg (BH). Pathways with a FDR value of less than 0.05 were considered significantly differential.

### 2.6. Identification of DEGs

In our study, LIMMA package [20] of R language was used to calculate the expression level of each gene between two groups to further identify DEGs. Next, all expression scores were converted into fold-changes (FC) with log⁡2 base (herein, this means log⁡FC). The log⁡FC for each gene was determined as log(postmenopausal) − log(premenopausal), and the distribution of the log⁡FC value of each gene expression was investigated. log⁡FC was on behalf of the differential expression degree. Then, we used multiple test to adjust the original *P* values by means of Benjamini et al. [21] method using FDR. DEGs were finally extracted when the threshold was set at FDR < 0.05 and |log⁡FC| ≥ 2.

### 2.7. Pathway Analyses for DEGs

To further reveal the biological roles of DEGs, DAVID [22] was used to perform the pathway analysis using the Expression Analysis Systematic Explorer (EASE) test [23]. The EASE score was utilized to extract the significant categories. In our work, the pathways having *P* < 0.05 were regarded to be significant pathways.

### 2.8. Comparison of iPAS and DAVID

To evaluate whether the iPAS method was feasible, the method proposed in the current study was compared with traditional DAVID software. The common pathways between the two methods were identified. In an attempt to evaluate the classification capacity of the common pathways, the hierarchical clustering analysis was implemented for the common pathways using Gitools [19].

### 2.9. TargetScore Calculation and Prediction of Potential miRNAs

In detail, TargetScan context score (TSCS) is a sequence-based score for the individual target site, which was computed using TargetScan [24]. Probabilities of conserved targeting (PCT) are the probability of conserved targeting for single target site [25]. TargetScore is a flexible package which takes log⁡FC values and sequence scores as inputs to calculate the probability of each gene being the target of a given miRNA. The function “TargetScore” offers a convenient way to acquire TargetScore with precomputed input (log⁡FC, TSCS, and PCT), and the range of TargetScore is from 0 to 1 [26]. The greater the TargetScore is, the higher the accuracy in detecting known targets is. Therefore, relying on the log⁡FC, TSCS, and PCT values, we counted the TargetScore for the genes enriched in the significant pathways to further identify the potential miRNAs by means of Variational Bayesian-Gaussian Mixture Model (VB-GMM) [27]. The TargetScore distribution for validated and nonvalidated targets of all miRNA-mRNA interactions each having at least 1 validated targets was evaluated. In the current study, the predefined *κ* was determined as the threshold when little overlap between the two distributions when TargetScore = *κ* existed. Furthermore, the miRNA-regulated pathways were achieved using their target gene sets. Next, we constructed an MRPN consisting of miRNAs, genes, and their common pathways. Cytoscape 2.8.3 was used to visualize the MRPN, and the topological properties of the MRPN were analyzed based on the Network Analysis plugin [28].

## 3. Results

### 3.1. Identification of Differential Pathways

**Figure 2:**
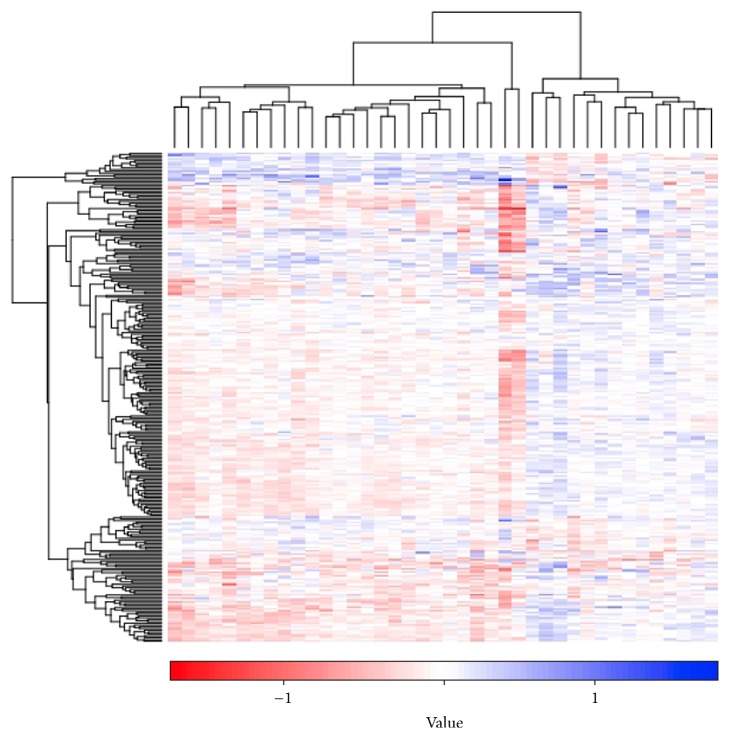
Cluster analysis of using average *Z* approach as the iPAS on the dataset of postmenopausal osteoporosis (PMOP) through Gitools method. Pathways (*n* = 279) and samples are clustered based on iPAS. The color scale represents the relative levels of pathway aberrance. Horizontal axis is samples; vertical coordinate is differential pathways. iPAS means individual pathway aberrance score.

After the gene-level statistics of all genes was transformed into the pathway-level statistics value of each pathway, *t*-test was used for measuring the pathway statistics of each pathway in control and PMOP samples. Moreover, FDR approach was applied to correct significance levels (*P* values) for multiple hypothesis testing. Using the criteria of FDR < 0.05, a total of 279 pathways were extracted between the two groups. Cluster analysis was conducted to explore the changes of these 279 pathways. The heatmap of the 279 pathways was shown in Figure 2.

### 3.2. Identifying DEGs

Before pathway analysis, DEGs between the two groups were firstly identified. Using the criteria of FDR < 0.05 and |log⁡FC| ≥ 2, a total of 39 DEGs were retrieved. Specific information about DEGs was exhibited in Table 1. The most 5 significant DEGs were* PTGES2*,* HAB1*,* SCT*,* ASNSD1*, and* TUT1*.

### 3.3. Comparison of iPAS and DAVID

To evaluate whether the iPAS method was feasible, the method proposed in the current study was compared with traditional DAVID software. It was observed that a total of 27 pathways were the common ones obtained from the iPAS approach and DAVID software. These common pathways were shown in Table 2. More importantly, hsa0410 (MAPK signaling pathway) was the first significantly enriched pathway. Of note, the pathway quantity selected from iPAS was increased, relative to the DAVID method (a total of 279 differential pathways were obtained by means of iPAS method, while only 64 significant pathways were extracted based on the traditional DAVID). Thus, our study demonstrated that the iPAS method showed more efficiency to identify significant pathways, compared with DAVID.

**Figure 3:**
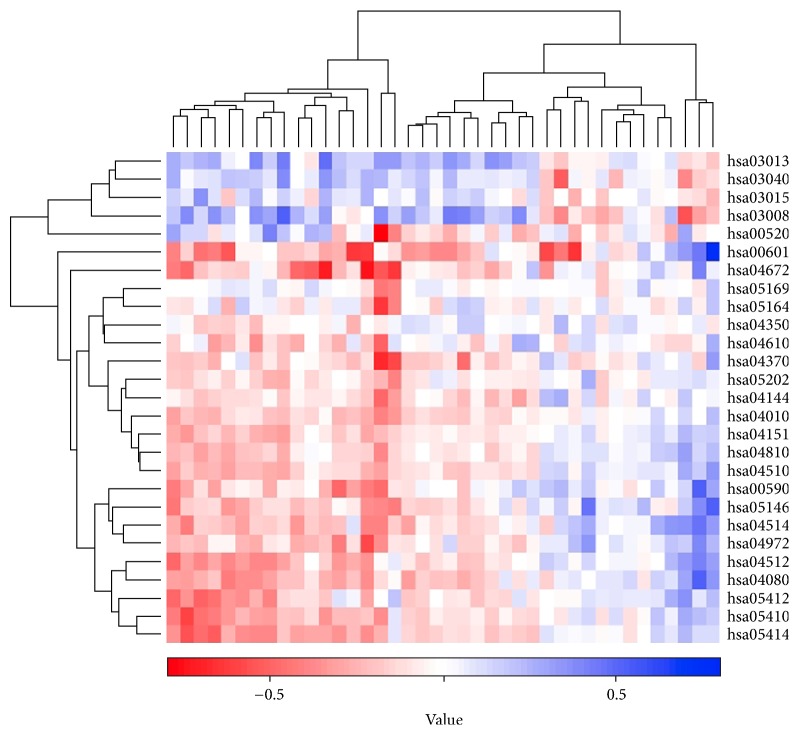
The cluster heatmap of the common pathways based on Gitools method. The color scale stood for the iPAS level; horizontal axis denoted samples; vertical coordinate represented pathways.

The clustering analysis was carried out to evaluate the classification capacity for microarray data samples using the common 27 pathways detected by iPAS and DAVID method (Figure 3). The classification efficiency for microarray data samples was computed based on accuracy. These pathways separated PMOP from controls with the accuracy of 0.912. In light of this, we further infer that these pathways could be used to be marker pathways for PMOP with a high accuracy.

### 3.4. Construction of MRPN for the Common Pathways Based on the Common Genes of miRNAs and Pathways

**Figure 4:**
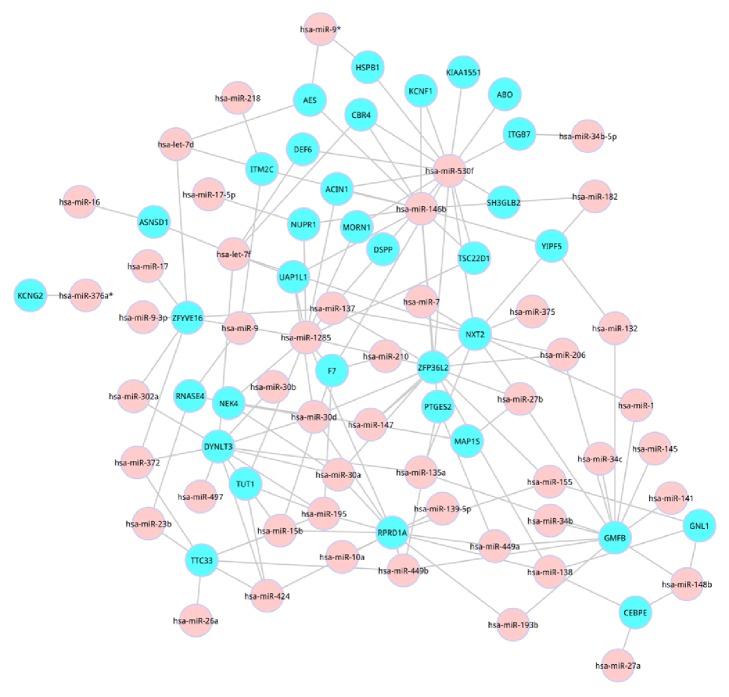
Relationships between the common genes in the common pathways and miRNAs.

TargetScores were firstly calculated based on the combination of log⁡FC values, and TSCS as well as PCT scores. Then, the distribution of TargetScores was obtained. According to the distribution of TargetScores, little overlap between the two distributions at TargetScore = 0.4 was found. Hence, TargetScore > 0.4 was selected as the threshold to filter out less confidence miRNAs. Using the TargetScore value > 0.4, a total of 39 genes, 50 miRNAs, and 145 miRNA-gene interactions were identified, and specific relationship was displayed in Figure 4.

**Figure 5:**
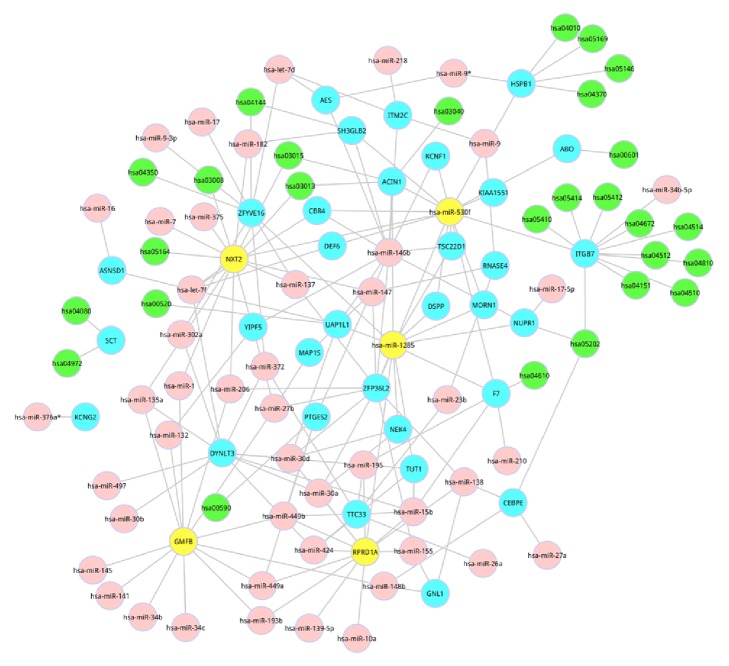
MiRNA-regulated pathway network (MRPN) in PMOP. Network organization of pathway, gene, and miRNA associations. Red nodes represented miRNAs, and blue squares as well as blue nodes indicated genes, pathways, respectively. Yellow ones were the hub nodes.

We then constructed an MRPN for PMOP, which was shown in Figure 5. In this network, the most significant common pathway (MAPK signaling pathway) was regulated by has-miRNA-9. Furthermore, has-miRNA-34b-5p and has-miRNA-530f indirectly regulated 10 significantly differential pathways; among these 10 pathways, hsa-04151 (PI3K-Akt signaling pathway) was the second most significant common pathway.

### 3.5. Topological Features of the MRPN

We determined the topological characteristics of the network, including the degree distribution. The degree distribution followed a power law distribution using all of the nodes in the MRPN. Based on the degree distribution, we selected 5 nodes as hub nodes, including NXT2, has-miR-1285, GMFB, has-miR-530f, and RPRD1A.

## 4. Discussion

In our study, a novel method of extracting pathway-based biomarkers in PMOP was developed, by investigating the interactions among genes, miRNAs, and pathways associated with pathogenesis through constructing an MRPN. The results of our work identified 279 differential pathways between PMOP and normal samples, including MAPK signaling pathway and PI3K/Akt signaling pathway.

MAPKs play crucial roles in cellular response to growth factors or cytokines [29]. The musculoskeletal system is frequently observed in several of the Ras/MAPK pathway syndromes, suggesting that activation of the Ras/MAPK pathway impacts cells regulating bone development and homeostasis [30]. OP is the systemic bone remodeling disease caused by imbalance between bone formation and bone resorption. Significantly, MAPK has been implicated to trigger the induction of c-Fos [31], and c-Fos is a key transcription factor of osteoclastogenesis [32]. Moreover, osteoclastogenesis exerts important functions in the development of OP [33]. MAPK signaling pathway has been demonstrated to be differentially activated in MSCs derived from osteoporotic postmenopausal women [34, 35]. Therefore, we infer that MAPK is important for bone formation and may represent a target to prevent bone loss associated with PMOP. Of note, this pathway was regulated by has-miRNA-9. Has-miRNA-9 was a proinflammatory miRNA [36]. A former study has demonstrated that has-miRNA-9 plays important roles in suppressing tumor growth via mediating the downstream p38 MAPK pathway [37]. Accordingly, has-miRNA-9 might play important roles in the PMOP progression, partially through regulating the MAPK signaling pathway.

Previous studies have suggested that PI3K-Akt signaling pathway is implicated in the regulation of cell growth, proliferation, and survival [38]. Osteoblastic bone formation is stimulated through activating the PI3K-Akt signaling pathway [39]. Bone remodeling is an essential physiological process regulating bone mass and strength; an imbalance in bone resorption and formation results in bone loss during aging and in OP. Moreover, PI3K/Akt pathway is very important in osteoclastogenesis and bone mass in mice, which may offer a potential target in OP [40]. Remarkably, osteoclastogenesis exerts important functions in OP progression [33]. Has-miRNA-530f as a hub node in the MDPN regulated this pathway of PI3K/Akt signaling pathway in our study. According to the literatures, no studies have reported the association of has-miRNA-530f and PMOP. However, based on our results, we speculate that PMOP might be related to has-miRNA-530f-regulated PI3K/Akt signaling pathway.

## 5. Conclusion

In conclusion, we comprehensively collected the PMOP risk pathways and the miRNAs of PMOP to characterize the molecular mechanism and treatment strategy for PMOP. We then constructed an MRPN to better reveal the relationship among genes, pathways, and miRNAs and to develop promising drug candidates for PMOP. Based on the results, differential pathways, including MAPK signaling pathway and PI3K/Akt signaling pathway, were successfully identified. Moreover, these pathways-related miRNAs may be involved in the pathogenic process of PMOP. The findings of our study may be applied clinically for the diagnosis and treatment of PMOP patients.

## Figures and Tables

**Table 1:** List of differentially expressed genes (DEGs).

Genes	Log (fold change)	False discovery rate (FDR)
PTGES2	2.300507	0.001222
HAB1	−2.30022	0.001222
SCT	−2.40399	0.001607
ASNSD1	−3.32248	0.002156
TUT1	2.102886	0.003676
SEPT5-GP1BB	−2.35928	0.004771
NXT2	2.887908	0.005497
ACIN1	−3.31279	0.005719
RPRD1A	4.450723	0.005802
GMFB	2.307129	0.005802
GNL1	3.411528	0.009374
MORN1	−3.39931	0.010842
SH3GLB2	2.502244	0.014611
NEK4	2.627295	0.018591
NUPR1	−2.38971	0.018607
AES	−2.20363	0.019621
DEF6	2.320463	0.022823
LOC100507630	2.187249	0.022839
ABO	3.329499	0.025143
KCNG2	−3.36086	0.028305
ITGB7	2.433329	0.029951
TTC33	−2.37332	0.030839
KCNF1	2.32550	0.035143
F7	−2.65548	0.035305
KIAA1551	−2.32991	0.035682
DSPP	2.341588	0.036731
RNASE4	−2.30475	0.036903
MAP1S	2.317352	0.037114
DYNLT3	−2.24231	0.038002
YIPF5	−2.31073	0.039739
ITM2C	−2.73745	0.040851
HSPB1	−2.52917	0.041666
ZFYVE16	2.43259	0.041951
UAP1L1	2.38412	0.045666
CBR4	2.32525	0.046791
TSC22D1	−2.61448	0.047839
HAMP	−2.36871	0.047872
ZFP36L2	2.31584	0.049019

**Table 2:** List of the common pathways between the two methods.

Pathways	DEGs
MAPK signaling pathway [PATH: hsa04010]	HSPB1
PI3K-Akt signaling pathway [PATH: hsa04151]	ITGB7
VEGF signaling pathway [PATH: hsa04370]	HSPB1
TGF-beta signaling pathway [PATH: hsa04350]	ZFYVE16
Transcriptional misregulation in cancers [PATH: hsa05202]	NUPR1, ITGB7, CEBPE
mRNA surveillance pathway [PATH: hsa03015]	NXT2, ACIN1
RNA transport [PATH: hsa03013]	NXT2, ACIN1
Glycosphingolipid biosynthesis-lacto and neolacto series [PATH: hsa00601]	ABO
Endocytosis [PATH: hsa04144]	SH3GLB2, ZFYVE16
Amino sugar and nucleotide sugar metabolism [PATH: hsa00520]	UAP1L1
Intestinal immune network for IgA production [PATH: hsa04672]	ITGB7
Arachidonic acid metabolism [PATH: hsa00590]	PTGES2
Complement and coagulation cascades [PATH: hsa04610]	F7
Arrhythmogenic right ventricular cardiomyopathy (ARVC) [PATH: hsa05412]	ITGB7
TGF-beta signaling pathway [PATH: hsa04350]	ZFYVE16
Ribosome biogenesis in eukaryotes [PATH: hsa03008]	NXT2
Hypertrophic cardiomyopathy (HCM) [PATH: hsa05410]	ITGB7
ECM-receptor interaction [PATH: hsa04512]	ITGB7
Dilated cardiomyopathy (DCM) [PATH: hsa05414]	ITGB7
Pancreatic secretion [PATH: hsa04972]	SCT
Amoebiasis [PATH: hsa05146]	HSPB1
Spliceosome [PATH: hsa03040]	ACIN1
Cell adhesion molecules (CAMs) [PATH: hsa04514]	ITGB7
Influenza A [PATH: hsa05164]	NXT2
Epstein-Barr virus infection [PATH: hsa05169]	HSPB1
Focal adhesion [PATH: hsa04510]	ITGB7
Regulation of actin cytoskeleton [PATH: hsa04810]	ITGB7
Neuroactive ligand-receptor interaction [PATH: hsa04080]	SCT
